# An Optimal Current Controller Design for a Grid Connected Inverter to Improve Power Quality and Test Commercial PV Inverters

**DOI:** 10.1155/2017/1393476

**Published:** 2017-04-30

**Authors:** Ali Algaddafi, Saud A. Altuwayjiri, Oday A. Ahmed, Ibrahim Daho

**Affiliations:** Electrical Electronics Engineering Department, Sirte University, Sirte, Libya

## Abstract

Grid connected inverters play a crucial role in generating energy to be fed to the grid. A filter is commonly used to suppress the switching frequency harmonics produced by the inverter, this being passive, and either an L- or LCL-filter. The latter is smaller in size compared to the L-filter. But choosing the optimal values of the LCL-filter is challenging due to resonance, which can affect stability. This paper presents a simple inverter controller design with an L-filter. The control topology is simple and applied easily using traditional control theory. Fast Fourier Transform analysis is used to compare different grid connected inverter control topologies. The modelled grid connected inverter with the proposed controller complies with the IEEE-1547 standard, and total harmonic distortion of the output current of the modelled inverter has been just 0.25% with an improved output waveform. Experimental work on a commercial PV inverter is then presented, including the effect of strong and weak grid connection. Inverter effects on the resistive load connected at the point of common coupling are presented. Results show that the voltage and current of resistive load, when the grid is interrupted, are increased, which may cause failure or damage for connecting appliances.

## 1. Introduction

Understanding the grid code requirements is very important in grid connected inverter. A grid connected inverter is a solar system that works in parallel with the grid [1]. The electricity that is generated by photovoltaic (PV) panels can be exported to the grid or used by the local load. A storage using, for example, batteries, is not included, with clear cost benefits over stand-alone renewable installations. PV generation is becoming increasingly widespread. According to the authors in [1, 2] a solar PV system is the third most important of renewable energy, after hydro- and wind power. Worldwide PV capacity was approximately 70 GW in 2011. Europe is the largest PV energy producer with capacity 51 GW, followed by Japan (5 GW), and then the USA (4.4 GW), China (3.1 GW), Australia (1.3 GW), and India (0.466 GW) [1, 2]. PV generation has been implemented in many developing countries. Those regions have a large subbelt and could have 1100 GW installed capacity by the year 2030 [3]. As PV generation capacity increases, guidelines or standards will be needed to govern the import and export of power to and from the grid, and their importance will increase. Many organisations are dealing with PV codes and safety standards such as the Institute of Electrical and Electronic Engineering standard (IEEE-1547) and the International Electro-Technical Commission standard (IEC-61727). In this paper, the grid connected inverter is used to connect solar panels to the grid. In other words, inverters form a crucial link in renewable energy systems between the generating components such as wind turbines, solar photovoltaic, and the rest of the grid. Full bridge DC/AC topologies are commonly used in grid connected inverters with a high switching frequency (e.g., 15000 Hz). The harmonics generated by high switching frequency, however, limit the efficiency of grid connected inverters. An LCL-filter is usually used to mitigate the limitations and satisfying required grid standards [4]. However, the virtual impedance of the grid network and the interaction between the main elements of the LCL-filter make designing an effective controller challenging, due to high peak gain at the resonant frequency. Some methods and techniques were developed to overcome this issue and to improve the steady state and the transient response of the grid connected inverter, including repeat control, proportional resonant, and deadbeat control [4–7]. Channegowda and John [8] used a damping resistor to reduce LCL-filter resonance; although this was successful, damping resistor power losses were high enough to affect inverter efficiency. The upshot being that at the time of wiring, the LCL-filter in this application still has poor dynamic performance.

PV inverters should have certain design features intrinsically included, such as Maximum Power Point Tracking (MPPT), anti-island, power factor correction, harmonic reduction, and fault ride through [4]. Solar PV generation offers clear environmental advantages, being almost completely pollution-free at the point of generation, and unlike some other forms of renewable generation is silent, making it eminently suitable for residential areas too [1]. Distributed generation using PV system is also advantageous in countries where traditional heavy grid infrastructure is not in place. The requirement for small distributed power generation systems is low cost, high efficiency, and tolerance for a wide range of input voltage variations. The inverter can be a single stage or multiple stages. Single stage inverters convert DC directly to AC with no intermediate stage. Two-stage inverters take the form of a DC-DC converter followed by a DC-AC inverter [9–12]. Single stage inverters offer simple structure and low cost, but multiple stage inverters can accommodate a wider range of input voltages. For multiple (e.g., DC/DC and DC/AC converter) stages inverters, complexity and cost are increased, and efficiency is lower [1, 9].

The L-filter is suitable for an inverter with a high switching frequency, this being a first-order filter with 20 dB/decade overall frequency. The dynamic interaction between inverters that use L-filters is less than it is between the inverters with LC- or LCL-filters. Also, variation in environmental conditions can result in the generation of a nonsinusoidal current to the network that may cause a voltage drop and distortion across it. Liang et al. presented voltage distortion analysis [13]; however, their study did not include analysis of current at the point of common coupling (PCC). The PV inverters are limited by several factors, including the weak grid connection, island mode connection, and the interaction between different PV inverters. The island mode connection can be dangerous to utility workers and may prevent utility interface with G59 protection to reconnect the network with the PV system. This requires developing an anti-islanding mode connection to stop producing power to the main grid. However, the anti-islanding mode connection may cause events of the power shortage (a blackout). A backup inverter can overcome this limitation. The backup inverter is used to supply the critical load and does not feed the grid. This is because the utility interface with G59 protection contains a Voltage Monitoring Detection (e.g., VMD460) relay, which is an external network and system protection. This relay is used to disconnect the public grid with the PV generator in events of unacceptable threshold values. The VMD460 relay is a device that monitors the grid voltage and frequency and when the voltage and frequency of the source energy are in the acceptable threshold, then the VMD460 relay allows reconnecting with the public grid. However, an interaction is likely to occur if multiple PV inverters are connected. Therefore, the AC Mini-Grid can be used to improve the performance of the system, which includes multiple PV inverters. Harmonic interference of large population of inverters was analysed by Enslin et al. [14]. The resonance between the PV inverter and existing network components was discussed. It was found that the output inverter impedance should be high since it is a function of frequency and it gives an unpolluted sinusoidal current waveform. The study assumed that most house appliances would be capacitive loads, and these tend to be inductive. Xue et al. provided an overview of future development trends for inverters [15]. Taking that case reliability improvements will be needed. It was mentioned that, in the future, the PV inverters are required to power ancillary service in the distribution grid. In general, PV inverter design criteria will include flicker mitigation, unbalanced compensation, active load balancing power, active filter, harmonic filtering, voltage sag, and swell mitigation control voltage. Anti-island detection was also identified as a challenge, and it should be possible to distinguish between permanent power outages and transient falls and provide an appropriate response in each case. The limitations of these studies were presented with critical reviews by Algaddafi et al. [16]. A new method to determine the inverter output impedance and grid impedance was presented by Sun [3], who argued that high output impedance for a grid connected inverter enables successful operation with a wider range of grid impedances. Other issues which would also be important include grid parallel inverter control, output filter and damping, the method of synchronising to the grid, and optimising output impedance.

In recent, a multiple inverter using Norton's equivalent circuits was modelled by He et al. [17], discussing the interaction of parallel inverters. The multiple PV inverter based Micro-Grid system presented a more challenging picture, where the PV inverters interaction will cause complex resonance at various frequencies, so the output current will be distorted even when the control design and filter circuit are accurately designed for a single inverter.

**Figure 1:**
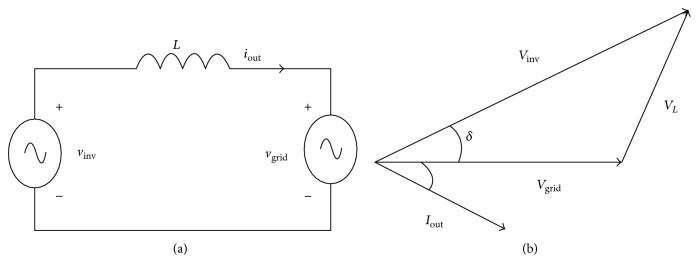
Grid connected inverter: (a) equivalent circuit and (b) phasor diagram.

A physical PV array has several limitations as it depends on the weather conditions for outdoor testing and requires a large space and cooling system for indoor testing. A PV array emulator (PVAE) with a fast response offers a potential solution. The currently available power electronic PVAEs have a slow response time. This can be overcome by using a series regulator with a robust control. The nonlinearity of the Current-Voltage (*I*-*V*) and Power-Voltage (*P*-*V*) curve generator of a PVAE is also challenging, and the characteristics of this will vary with solar insolation, ambient temperature, and output voltage [12]. This can be overcome by using an analogue computation circuit as presented in [18]. However, the PVAE that presented in [18] requires a cooling system, where with high load or high power the system may be unstable and the performance of PVAE will deteriorate. Therefore, the PVAE is unsuitable to operate Sunny Boy (SB 1700E) at the present time, even though it can be used with small power such as Sunny Boy 700 inverter. For the simplicity and availability, the commercial PV inverter, which is SB 1700E inverter, was initially tested with a Thevenin source in this paper. The operation of the single phase inverter is described as an equivalent circuit of the grid connected inverter, which helps to understand the behaviour of the PV inverter. The dynamic model of the inverter does not appear to be adequately discussed to date. Equation (1) is commonly used to study the behaviour of the PV inverter and to find the phase angle [19].

(1) $$\begin{array}{c} \left|\;V_{L}^{2}\;\right|=\left|\;V_{inv}^{2}\;\right|+\left|\;V_{grid}^{2}\;\right|-2\left|\;V_{grid}\;\right|\left|\;V_{inv}\;\right|\cos\delta , \end{array}$$

where *V*_*L*_ is the voltage drop on the inductor, *V*_inv_ is the inverter voltage, *V*_grid_ is the grid voltage, and *δ* is the load angle.  Figure 1 shows the equivalent circuit of the inverter connected to the main grid and a phasor diagram of the grid connected inverter. The phasor diagram is used to explain the circuit performance of the PV inverter. When the load angle (*δ*) is equal to zero, the grid voltage is equal to the output inverter voltage. Thus, the active power will be zero. Therefore, the amplitude of the inverter voltage should be higher than the utility network voltage. The load angle (*δ*) is used to control the active power (*P*) and the reactive power (*Q*) injected into the grid per (2) and (3) below [20].

(2) P=VgridwLVinvsin⁡δ

(3) Q=VgridwLVinvcos⁡δ−Vgrid

(4) VL=jwLIo,

where *w* = 2*πfL* is the reactance of the inductor connected between the grid and the inverter. The power factor (PF), which is cos⁡*φ*, is affected by three elements including the load angle, the inverter voltage, and the voltage drop on the inductor.

*V*_inv_sin⁡*δ* = *V*_*L*_cos⁡*φ*; if the PF approaches one, known as the unit PF, the output voltage and the output current will be in phase. This analysis assumes no losses in switching power electronic, which means ideal switches. Many inverters intend to operate at unity PF. The modulation index ratio could be calculated by dividing the output inverter voltage by the DC input voltage. The modulation index (*m*) is defined as the ratio between the fundamental components of the inverter voltage (*V*_inv_) and the DC input voltage of the inverter (*V*_DC_). This can be given by [18]

(5) $$\begin{array}{c} m=\frac{V_{inv}}{V_{DC}}. \end{array}$$

The phase difference between *V*_inv_ and *V*_grid_ is the angle *δ*. The inverter voltage and the load angle can be estimated by using (6) and (7), respectively, under the condition of inverter work at unity power factor [18].

(6) Vinv=VL2+Vgrid2

(7) δ=tan−1⁡VLVgrid.

This mathematical approach and theory will help to design open loop grid connected inverter. To summarise, in general, the harmonic components are required to be filtered whether by using the passive filter or the active filter. The latter uses extra switches and may bring extra loss and also it is a complex [21–23]. The passive filters could be L-filter, LC-filter, LCL-filter, and LLCL-filter. The higher order such as LCL-filter or LLCL-filter may suffer from the resonance oscillation [21, 24–26]. Therefore, in order to overcome the above limitations, this paper presents a simple method to design an optimal control of a grid connected inverter with an L-filter. It is organised as follows: a configuration and the numerical modelling of the grid connected inverter with optimal design control are discussed in Section 2. In addition, Section 2 presents the simulation and experimental setup to investigate the grid connected inverter for a weak grid compared to a strong grid connection.  Section 3 discusses the acquired numerical and experimental results. The last section draws the conclusions and future work.

## 2. Configuration and Modelling of the Grid Connected Inverter

It is necessary to consider the requirements of the grid and how a single connected inverter interacts with this. A mathematical modelling of a grid converter, which was followed by designing an optimal controller to improve overall power quality, was developed. Experimentation for this work was carried out using a commercial PV inverter; in this case, the Sunny Boy 1700E inverter was utilised.

### 2.1. Simulation Part

The simulation section includes a numerical model of grid connected inverter with open loop circuit and then designing the optimal controller of the same circuit of grid connected inverter.

#### 2.1.1. Grid Connected Inverter in the Open Loop Circuit

The inverter can be configured as a stand-alone inverter supplying a local load, a grid connected, or a bidirectional inverter. The grid connected inverter is considered in this paper. The utility network is defined as the infinite busbar, which has a constant voltage and a constant frequency. The grid synchronisation is very necessary for the grid connected inverter. It can be achieved by using a phase locked loop [4]. In this study, the desired current *I*_ref_ is given in

(8) $$\begin{array}{c} I_{ref}=12\sin\left(\;wt\;\right) \end{array}$$

The open loop control of the grid connected inverter was modelled theoretically and simulated in Simulink according to the assumptions listed in Table 1 with the theory described in the previous section. The voltage drop on the inductor* L* was calculated by (4). The modulation index was determined by dividing the inverter voltage by the DC input voltage according to (5). The inverter voltage was estimated by (6). Load angle was calculated by (7). The outcomes of these equations are as follows: the modulation index = 0.658, the load angle *δ* = 6.59°, and the inductor voltage *V*_*L*_ = 37 V.

**Figure 2:**
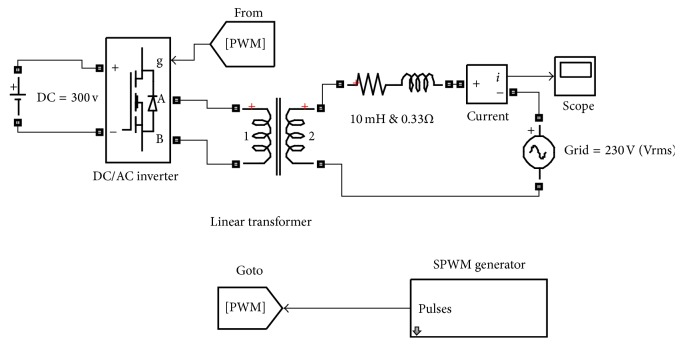
Open loop circuit diagram of the DC/AC grid connected inverter.

These results were calculated theoretically; thus the simulation model should provide the same results to validate the model. The modelling of the power circuit consists of a constant DC source, a universal bridge, an L-filter, a linear transformer, and an AC voltage source. The linear transformer has two features: Firstly, it provides galvanic isolation of the DC components, and secondly, it can boost the voltage level to match the grid voltage. The control circuit used a pulse width modulation generator block, which was inserted into the modulation index, the carrier frequency, the phase angle *δ*, and the output grid frequency. The predicted inverter output voltage and the inductor voltage were acquired between terminals A and B of the universal bridge as shown in Figure 2. By using Fast Fourier Transform (FFT) analysis the fundament inverter voltage was found to be 197 V, whereas the inductor fundamental voltage was found to be 37 V.

**Figure 3:**
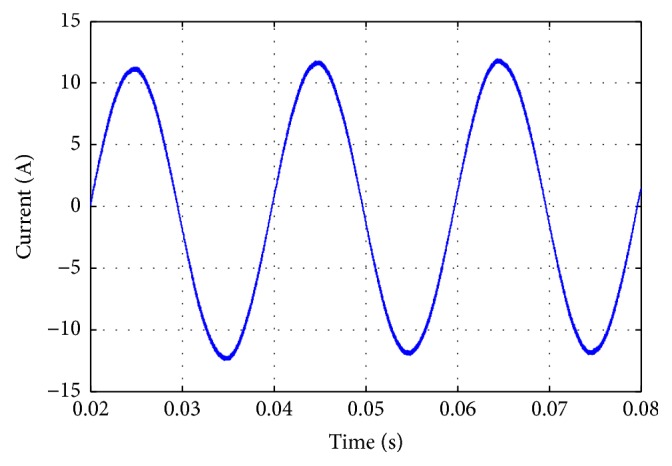
Output current of the DC/AC grid connected inverter.

Figure 3 shows the predicted output current of the inverter where the current value is nearly 12 A and the frequency is 50 Hz. This can validate the open loop control of the grid connected inverter, where the output current is symmetrical to the assumed reference current.

The following section focuses on the design of the control system for the grid connected inverter using the single-input, single-output in MATLAB. This is done by assuming ideal switching of the full bridge converter, where the L-filter with its parasitic resistance is used to design the optimal compensator.

#### 2.1.2. Proposed Grid Connected Inverter with L-Filter and Feedback Controller

**Figure 4:**
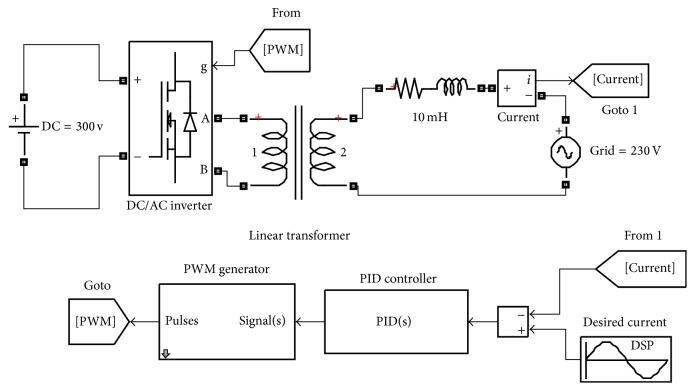
DC/AC grid connected inverter with L-filter used a unit proportional gain controller.

Here the grid connected inverter along with its controller design is modelled numerically and Proportional-Integral-Derivative (PID) methods are investigated. The aim is to find the optimal controller method by observing the resultant total harmonic distortion (THD).  Figure 4 shows the modelled grid connected inverter with a feedback controller. The output current was compared with the reference current and then passed through the PID controller block. The reference current was set to 12 A at 50 Hz and the phase offset was set to zero. In the same manner, the grid voltage, the frequency, and the phase offset were set to 230 V, 50 Hz, and 0, respectively. As a result, the reference current is in phase with the grid voltage. It is assumed that the grid is a strong grid with no harmonic generated by the grid impedance.

#### 2.1.3. L-Filter

An L-filter is suitable for inverters, which have high switching frequency. It can be designed easily according to

(9) $$\begin{array}{c} P_{o}=\frac{V_{grid}}{X_{L}}\sqrt{V_{dc}^{2}-V_{grid}^{2}}, \end{array}$$

where *P*_*o*_ is the output power, *V*_dc_ is the voltage DC link, *V*_grid_ is the grid voltage for the single phase, and *X*_L_ is the reactance of the L-filter, which determines the value of the inductor according to the switching frequency. The ripple current injected into the grid must be less than 3% of the rated current.

**Figure 5:**
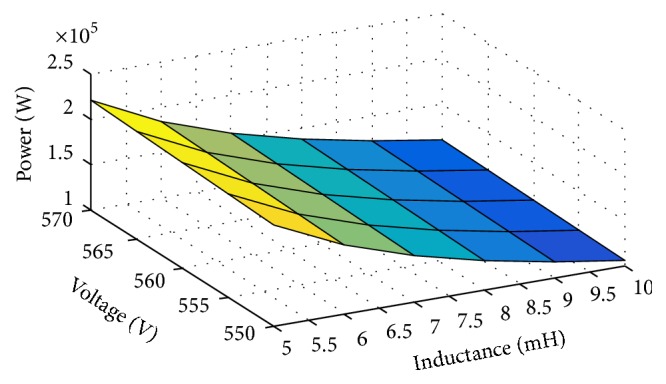
Characteristics of the L-filter.

An L-filter can be readily optimised according to (9). The impact of variation of DC voltage and inductor on the power of the inverter is shown in Figure 5, where this figure presents the output power as a function of DC voltage and inductor inductance. Therefore, the selected L-filter is a trade-off between the harmonic required, cost, and required output power.

### 2.2. Experimental Part

The experimental work presents a simple procedure to test a commercial PV inverter and impact of interrupt network and SB 1700E inverter on the resistive load along with testing the speed response of PV inverter.

#### 2.2.1. Requirements to Test the SB 1700E Inverter Experimentally and Testing a Weak Grid Connection

**Figure 6:**
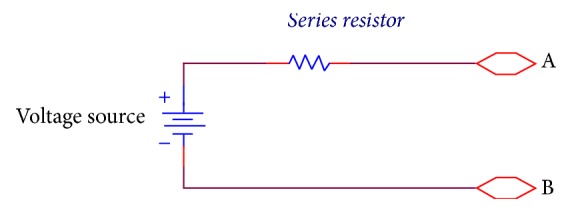
Thevenin source implemented to test SB 1700E inverter.

A PV inverter requires the real PV array and the main grid. However, the use of the real PV array in the laboratory may be impractical due to limitations mentioned above. Ideally, a PVAE is required to operate the PV inverter at the maximum power point (MPP). A Thevenin voltage source was initially used to test SB 1700E inverter. Thevenin source is a voltage source and a series resistor as shown in Figure 6. The PV inverter can be connected between the terminals A and B in order to test the inverter experimentally.

The SB 1700E inverter was connected to the Thevenin source to investigate the behaviour of the inverter and also this experiment aimed to understand the equipment performance and connection devices such as the series resistance (*R*_*s*_), DC supply voltage, and the power system analyser (PA2100). The SB 1700E inverter was tested when the input voltage, *R*_*s*_, and the additional inductor line were varied. According to the manual description of the SB 1700E inverter [27], when the grid impedance is higher than 1.25 Ω, or the grid voltage exceeds the range between −15% and +10% of the nominal grid voltage or the grid frequency exceeds the range of 0.2 Hz, the SB 1700E inverter is disconnected from the main grid within 0.2 sec. Inverter limitations were tested experimentally as follows: A variable inductor was used to create a weak grid. The weak grid could also be achieved by using a synchronous generator with a VAR controller. However, it may be regarded as an overly expensive method. Hence, the variable inductor is used to create a weak grid. SB 1700E inverter documentation states that if the line impedance exceeds 1.25 Ω, the inverter will switch off. Thus, when the reactance of the additional inductor is greater than 1.25 Ω, then the grid connection is referred to as a weak grid connection. Strong grid connection tests were carried out first, followed by weak grid experiments.

**Figure 7:**
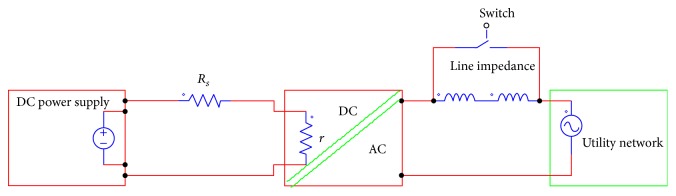
Testing the impact of the weak grid connection on the SB 1700E inverter by adding an additional inductor.

The SB 1700E inverter has an internal resistance (*r*) and it is operated in the range of DC voltage 139–400 V, the nominal operating voltage is 180 V, and the maximum DC current is 12.6 A. The AC side (grid connected) has 230 V operating voltage, 1500 W power, 8.5 A maximum output current, and 50 Hz nominal frequency. In order to test the PV inverter with the DC supply voltage, *R*_*s*_ is used. *R*_*s*_ contains 3 banks (a, b, and c), each consisting of 8 resistors connected in parallel, each resistor being switchable in or out of the circuit as appropriate, and each rated at 228 Ω and 250 W. The conducted experiments used eight resistors connected in parallel to give 28.5 Ω or 12 resistors to give 19 Ω. Apparatus consisted of a DC source supply, series resistor banks, a commercial inverter such as the SB 1700E inverter, a Power Analyser (PA2100), and a switch connected in parallel to the additional inductor as shown in Figure 7, to toggle between the weak and strong grid connections.

Weak grid connection may cause instability, increase the harmonic components, and cause distortion in the output waveforms. The DC source voltage was set to 300 V and the inductor was varied and observed.

The weak grid has properties of a low short circuit power and high grid impedance. The Short Circuit Ratio (SCR) is given by [23]

(10) $$\begin{array}{c} SCR=\frac{S_{SC}}{S_{n}}, \end{array}$$

where *S*_*n*_ is the rated power of the PV inverter and *S*_SC_ is the short circuit at the PCC. The grid is considered as a strong grid when the SCR is above 20 and as a weak grid when the SCR is below 10 according to Etxegarai et al. [28].

#### 2.2.2. Impact of the Network Interruption with the SB 1700E Inverter on the Resistive Load

**Figure 8:**
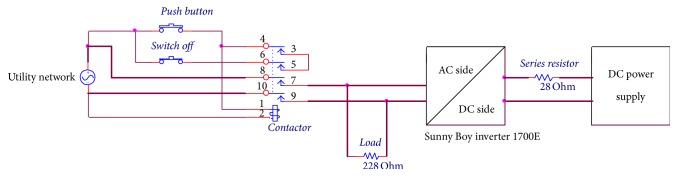
Circuit diagram of the SB 1700E inverter connected in parallel to grid and load 228 Ω.

The aim of this experiment is to test the impact of the SB 1700E inverter on the resistive load when the grid is interrupted. Experimental setup of this test is as follows: the push button momentary was used to connect the network distribution with the SB 1700E inverter and supplied the resistive load, while the normal close switch was used to disconnect the system from the main grid. The contactor 5.5 kW was used along with the momentary switch and normal close switch. A DC source voltage was connected to the SB 1700E inverter via a series resistance. A resistive load value of 228 Ω was connected to inverter output, which was connected in parallel to the main grid through the contactor as shown in Figure 8. The results of this experiment are presented in Section 3.2.2.

#### 2.2.3. The Response of the SB 1700E Inverter to Change of the MPP

The PV inverter needs to disconnect from the network in cases of abnormal grid conditions in terms of the voltage and the frequency. This provides safety of the utility maintenance workers and public network and avoids damaging elements of the PV system which are connected to the PV system [4]. This function of the PV inverter can be used to test the response of the SB 1700E inverter experimentally.

**Figure 9:**
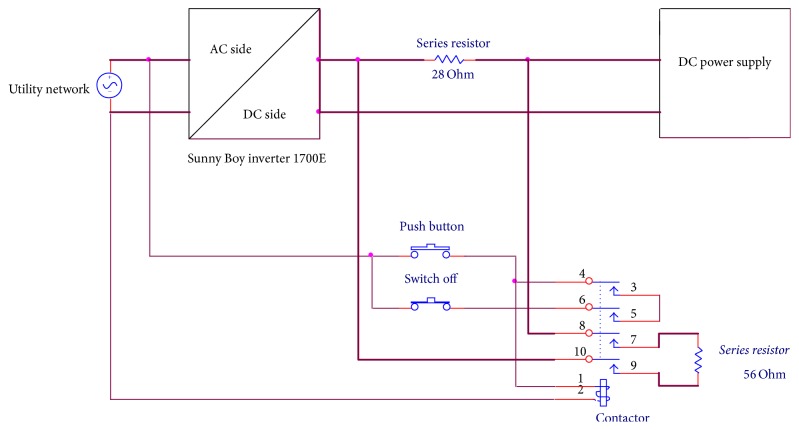
Circuit diagram for testing the MPPT and the speedy response of the SB 1700E inverter.

This test was used to find the response of the SB 1700E inverter to the change in the series resistor. This can help to design the PV array emulator in accordance with the response of the SB 1700E inverter. The series resistor 28 Ω was permanently connected in series between the DC source voltage and the DC side of the inverter as shown in Figure 9. An additional resistance value was 56 Ω, which was connected in parallel with the series resistor 28 Ω via a momentary switch and a contactor. The DC source voltage was set to 300 V and the oscilloscope (MDO3024) was triggered for 2 sec. The results and discussion of this test are presented in experimental work results.

## 3. Results and Discussion

Numerical model results are presented first in this section, followed by experimental work results. In the numerical model of grid connected inverter, an FFT analysis was used to evaluate the performance and efficiency of the proposed control system.

### 3.1. Simulink Results

**Figure 10:**
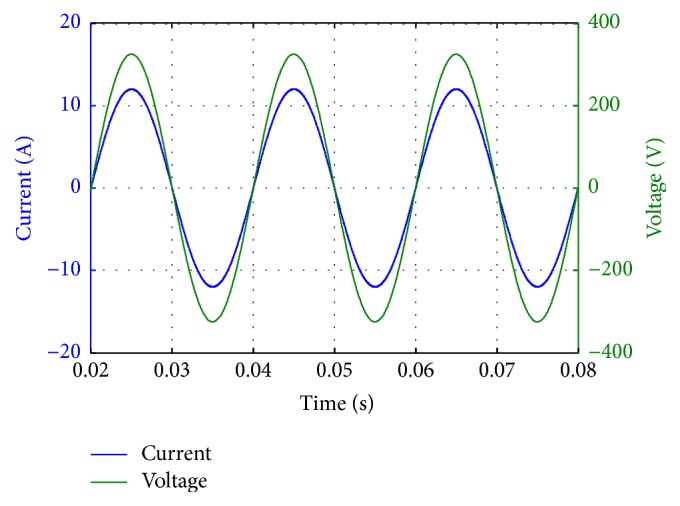
Output voltage and current of the grid connected inverter works at unity power factor.

In this section, the acquired results are displayed and discussed in detail.  Figure 10 depicts the grid voltage and the output current of the modelled grid connected inverter. It can be seen that the voltage and current are in phase. Thus, the modelled inverter operates at unity power factor.

**Figure 11:**
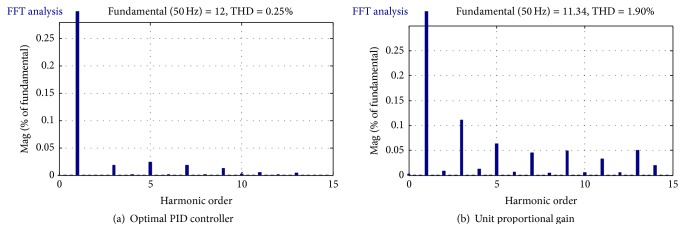
FFT analysis of the output current of the grid connected inverter and its frequency spectrum as in (a) and (b).

Figure 11(b) illustrates the waveform of the modelled inverter output current and its FFT analyses. Using a unit proportional gain controller, the output current decreased from 12 A to 11.3 A, although some ripple remains which could be removed. Even though the THD is larger than the optimal controller, it is still acceptable and does satisfy the required IEEE-1547 standard. An optimal controller eliminates the current ripple and improves the output current waveform as depicted in Figure 11(a). In the case where the unit proportional gain controller is used, the third current harmonic is the dominant harmonic, while the fifth current harmonic is the dominant harmonic when the optimal controller was modelled. It can be seen that the optimal current controller has reduced the current harmonic value by 1.65%. The proportional gain of an optimal current controller was found 18, the integral gain was found 22232.4, while the derivative was set to zero.

Due to the nonlinearity of switching power electronics of the grid connected inverter, a trial and error (manual) methodology was adopted by Elsaharty and Ashour [29] to obtain the value of the L-filter and the LCL-filter to comply with the standard IEEE-1547. Their acquired filter value was very large, which will increase the cost and size of the inverter. Therefore, this paper proposes a control method to improve the efficiency of the grid connected inverter, with a reduced cost and size.

### 3.2. Experimental Results

Acquired results of testing a commercial PV inverter are given as follows.

**Figure 12:**
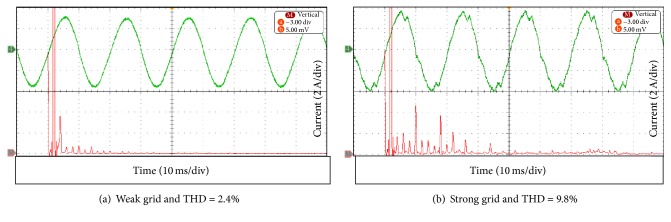
Output current waveform and its FFT analysis at varying inductor as in (a) and (b).

The AC side was directly connected to the grid without an additional inductor. The connected DC source voltage varied between 150 and 300 V in cases where the series resistance is *R*_*s*_ = 28 Ω, as listed in Table 2. Those results are measured by Power Analyser System (NORMA D6000). This table is used to describe the behaviour of SB 1700E inverter. The fifth current harmonic in Figure 12(b) is the dominated harmonic; on the other hand, when an additional inductor was increased the current harmonic was reduced as shown in Figure 12(a). This occurs because the additional inductor acts as a filter for the output current. This improves the current waveform.

**Figure 13:**
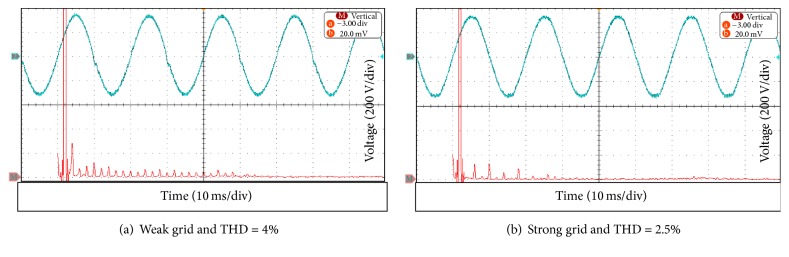
Output inverter voltage waveform and its FFT analysis as in (a) and (b).

The parameters of frequency and voltage setting of the SB 1700E inverter were changed to values different from the default values. The voltage range varied in 180–260 V and the frequency range ±4.5 Hz (starting from 50 Hz). This has been done to allow the SB 1700E inverter to operate with a bidirectional inverter (SUNNY ISLAND6.0H inverter), which will design a novel AC Mini-Grid system. The THD of the output current was measured using the Power Analyser (PA2100).  Figure 13 depicts the output voltage waveform of the SB 1700E inverter along with its FFT analysis at the weak grid and the strong grid connections. The third voltage harmonic is dominant for the harmonic in the case of the weak grid connection. The THD of the output voltage at the strong grid connection was 2.5%. The SB 1700 inverter voltage for the weak grid has a distinct distortion near the voltage zero crossing as shown in Figure 13(a).

Table 3 depicts the total harmonic distortion of the output voltage and current of the SB 1700E inverter at three different levels of DC source voltage 300 V, 200 V, and 160 V for both the strong grid and the weak grid connection. It can be seen that, at 160 V, the total harmonic distortion is roughly 47% and the PF is reduced. This will not meet with the IEEE-1547 standard [4]. Therefore, the grid connected inverter requires further study, where next publication will propose a new system that can be integrated with existing PV inverter to improve power quality of old PV inverter such as Sunny Boy 700 inverter and Sunny Boy 1700E inverter. This system is called AC Mini-Grid system that includes a bidirectional inverter with battery storage.

#### 3.2.1. Network Interruption of the SB 1700E Inverter with the Resistive Load

**Figure 14:**
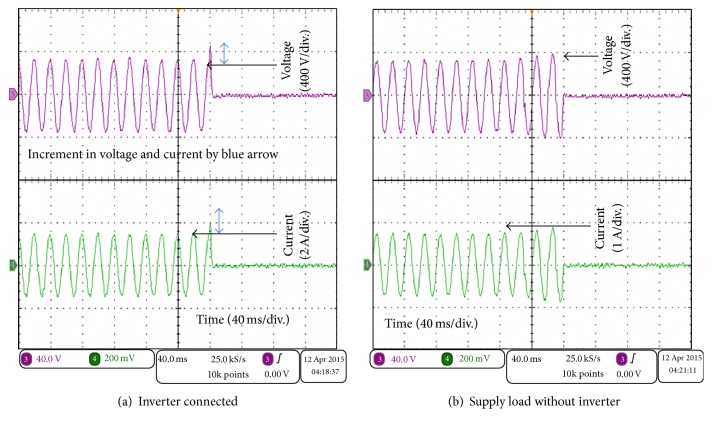
Waveforms of the load voltage in pink colour [10 V/Div.] and the current in green colour [10 A/Div.].

The result was obtained by using a Tektronix oscilloscope (MDO3024). The voltage and the current on the resistive load were measured in two methods. The first test is when the SB 1700E inverter was connected to the main grid, which was interrupted. Second test is when the main grid supplied the resistive load without connecting to the SB 1700E inverter. The comparison between these two connections was shown in Figure 14.

The results show that the resistive load connected to the main grid with the inverter has a significant effect on the measured voltage and current as shown in Figure 14(a). In both cases, the load was 228 Ω and the grid voltage was 230 V; thus the current is approximately 1 A. However, when the inverter was connected to the main grid and then the main grid was interrupted, the load voltage increased over the rated voltage and the load current increased over the rated current.

This test provides a framework for the exploration of the effect of the SB 1700E inverter on the resistive load. It was interesting that the voltage and the current waveforms sharply increased overrating of the voltage and the current. This increment in the voltage and the current may damage the connected appliances. The explanation of this increment in the load voltage and current could be due to the transformer or the filter of the SB 1700E inverter or its properties.

#### 3.2.2. Results of SB 1700E Inverter to Change of the MPP

**Figure 15:**
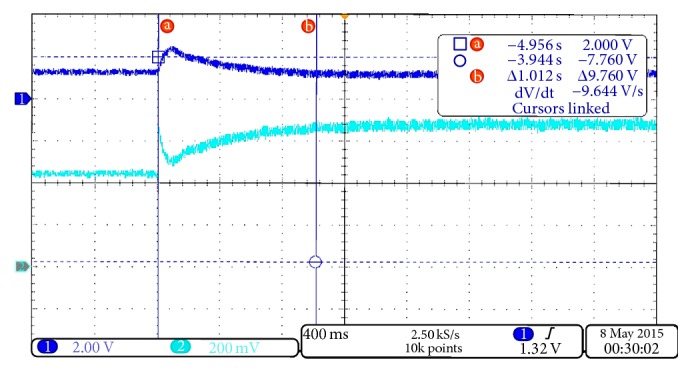
Input voltage trace 1 [200 V/Div.] and input current in trace 2 [10 A/Div.] at connecting the additional resistor.

The input voltage and current of the SB 1700E inverter were observed using the oscilloscope. The results were shown in Figure 15. The results show that the voltage is disturbed by connecting the additional series resistor and taking approximately 1.012 sec to settle to the level of the voltage. However, the input current was changed according to the change in the resistive load and has also taken 1.012 sec in both cases of the additional series resistor, connected and disconnected. Therefore, the SB 1700E inverter requires a PV array emulator with a fast response higher than 1.012 sec, in order to study the performance and behaviour of MPPT of PV inverter.

**Figure 16:**
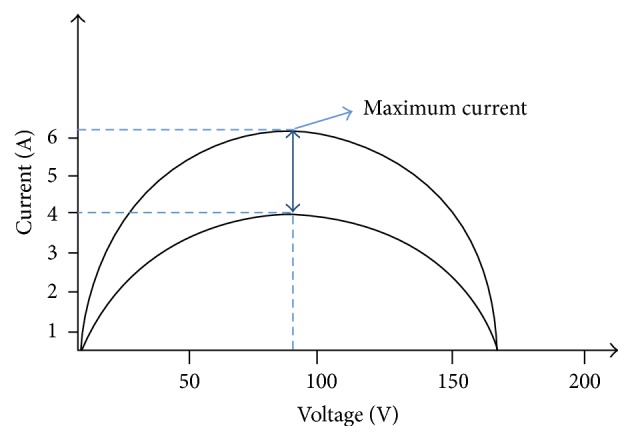
The response of the SB 1700E inverter to change of the additional series resistor.

Figure 16 shows the response of the SB 1700E inverter to change the series resistor from 20 Ω to 28 Ω and vice versa. The open voltage may not change when the series resistance was changed, but the voltage at the maximum power point has a small change. Perhaps the SB 1700E inverter may work in the line between the open voltage and the maximum power point. Consequently, the input current has a large variance according to the variation of the additional series resistor. The MPP is produced by multiplying the input voltage and current. The curve represented the maximum power as shown in Figure 16.

## 4. Conclusion

The paper studies a method to reduce the signal distortion using L-filter and PID feedback control technologies. A detailed review of the technologies is performed. The characteristics of a commercial inverter (SB 1700E) are also studied; besides this paper describes the behaviour of SB 1700E inverter. A grid connected inverter has been modelled with an L-filter in the open loop circuit. The optimal controller of the inverter helps to attenuate the harmonic components. In addition, the size of the filter with a good design controller can be reduced with the PID controller, but it has been selected to be 10 mH to easily describe the open loop circuit. This model can be used to connect any DC link inverter to the utility network. In addition, the proposed model can be used to connect a storage battery, which is charged by the renewable energy to the grid. A simple procedure to test a Sunny Boy 1700E inverter is developed. The impact of the weak grid connection has been investigated by implementing the variable inductor that is available at the laboratory of the Department of Engineering at the University of Leicester. It can be concluded that when the additional inductor value increases, the output voltage distortion increases and the current improves as the harmonic distortion is reduced. The speedy response of a commercial PV inverter is determined. Future work will focus on improving the power quality and the stability of the solar PV system in rural areas and design work for solar PV systems with attached storage that can improve the reliability and efficiency. The future work will also be considered designing a superior controller for the nonlinear model of the grid connected inverter with parasitic elements that were presented by Algaddafi in [30].

## Figures and Tables

**Table 1:** Assumption to model grid connected inverter.

Electrical parameters	Value
DC voltage source	300 V
AC voltage source	230 V, 50 Hz
Switching frequency	15 kHz
Inductor	10 mH
Output current	12 A
Parasitic resistance	0.33 Ω
Turn ratio of linear transformer	0.6

**Table 2:** System configuration acts as a strong grid to test the performance of the SB 1700E inverter under varying the DC voltage.

Number	Actual *R*_*s*_	Internal resistor of inverter	Reading *R*_*s*_ Ω	*V*dc (V)	*I*1 (A)	*V*1 (V)	*P*1 (W)	*I*2 (A)	*V*2 (V)	*P*2 (W)	*S*2 (VA)	Efficiency	P. F
				DC side				AC side					
1	31.5	32.3	28	300	4.7	152	727	2.9	229	675	689	0.93	0.98
2	31.0	35.7	28	280	4.2	150	634	2.6	229	590	601	0.93	0.98
3	32.0	35.1	28	260	3.87	136	544	2.2	228	500	510	0.92	0.98
4	31.2	41.5	28	240	3.3	137	451	1.9	228	420	430	0.93	0.98
5	31.1	50.4	28	220	2.7	136	369	1.57	228	340	350	0.92	0.97
6	32.0	68.0	28	200	2	136	278	1.2	228	250	280	0.9	0.89
7	33.8	104.6	28	180	1.3	136	182	0.87	228	160	206	0.88	0.78
8	35.9	214.1	28	160	0.64	137	88	0.6	228	70	140	0.8	0.50
9	0	0	28	140	The inverter switches off								

**Table 3:** THD of the output voltage, current, and PF of the SB 1700E inverter.

Total harmonic distortion (THD) (%)	300 VDC	200 VDC	160 VDC
Additional inductor			
Current	7.4	20.03	47.8
Voltage	2	2.18	2.4
Power factor (PF)	0.994	0.91	0.54
Without additional inductor			
Current	5.9	18	44
Voltage	2.4	2.35	2.2
Power factor (PF)	0.996	0.91	0.54

## References

[1] Alajmi B. (2013). *Design and control of photovoltaic systems in distributed generation [Ph.D. thesis]*.

[2] EPIA Global market outlook for photovoltaics until 2016. http://www.solarify.eu/wp-content/uploads/2012/05/EPIA_Global-Market-Outlook-2016.pdf.

[3] Sun J. (2011). Impedance-based stability criterion for grid-connected inverters. *IEEE Transactions on Power Electronics*.

[4] Teodorescu R., Liserre M., Rodríguez P. (2011). *Grid Converters for Photovoltaic and Wind Power Systems*.

[5] Leong T. T., Ishak D. Deadbeat-based PI controller for stand-alone single-phase voltage source inverter using battery cell as primary sources.

[6] Cao F., Li W., Wu J., He X. Effect of connection cable impedance on multi-inverter parallel system and an optimized controller with zero steady circulating current.

[7] Yao W., Lu Z., Long H., Li B. Research on grid-connected interleaved inverter with L filter.

[8] Channegowda P., John V. (2010). Filter optimization for grid interactive voltage source inverters. *IEEE Transactions on Industrial Electronics*.

[9] Chang Y.-H., Lin Y.-K. A closed-loop high-gain switched-capacitor-inductor-based boost DC-AC inverter.

[10] Lima J. C., Corleta J. M., Medeiros A. A PIC controller for grid connected PV system using a FPGA based inverter.

[11] Samerchur S., Premrudeepreechacharn S., Kumsuwun Y., Higuchi K. Power control of single-phase voltage source inverter for grid-connected photovoltaic systems.

[12] Datta A., Bhattacharya G., Mukherjee D., Saha H. (2014). An efficient technique for controlling power flow in a single stage grid-connected photovoltaic system. *Scientia Iranica*.

[13] Liang J., Zhou X., Jin X., Tong Y., Cai R. Analysis of voltage distortion for parallel PWM inverters with L filter.

[14] Enslin J. H. R., Hulshorst W. T. J., Atmadji A. M. S. Harmonic interaction between large numbers of photovoltaic inverters and the distribution network.

[15] Xue Y., Divya K. C., Griepentrog G., Liviu M., Suresh S., Manjrekar M. Towards next generation photovoltaic inverters.

[16] Algaddafi A., Elnaddab K., Khoja M. Review: grid connected inverter with its filter and providing suggestions for designing transformerless inverter.

[17] He J., Li Y. W., Bosnjak D., Harris B. Investigation and resonances damping of multiple PV inverters.

[18] Algaddafi A., Brown N., Gammon R., Altuwayjiri S., Rahil A., Ali S. An analogue computation based photovoltaic emulator for realistic inverter testing.

[19] Hassaine L., Olías E., Quintero J., Barrado A. Digital control based on the shifting phase for grid connected photovoltaic inverter.

[20] Mehta V. K., Mehta R. (2005). *Principles of Electrical Engineering*.

[21] Ashtinai N. A., Azizi S. M., Khajehoddin S. A. Control design in *μ*-synthesis framework for grid-connected inverters with higher order filters.

[22] Huerta F., Pizarro D., Cóbreces S., Rodríguez F. J., Girón C., Rodríguez A. (2012). LQG servo controller for the current control of LCL grid-connected Voltage-Source Converters. *IEEE Transactions on Industrial Electronics*.

[23] Ahmed K. H., Massoud A. M., Finney S. J., Williams B. W. (2011). A modified stationary reference frame-based predictive current control with zero steady-state error for LCL coupled inverter-based distributed generation systems. *IEEE Transactions on Industrial Electronics*.

[24] Twining E., Holmes D. G. (2003). Grid current regulation of a three-phase voltage source inverter with an LCL input filter. *IEEE Transactions on Power Electronics*.

[25] Huang M., Wang X., Loh P. C., Blaabjerg F. (2016). Active damping of LLCL-filter resonance based on LC-trap voltage or current feedback. *IEEE Transactions on Power Electronics*.

[26] Wu W., Sun Y., Huang M. (2014). A robust passive damping method for LLCL-filter-based grid-tied inverters to minimize the effect of grid harmonic voltages. *IEEE Transactions on Power Electronics*.

[27] Sunny Boy 1700E, Technical Description, http://files.sma.de/dl/5671/SB1700E-11-EE4403.pdf

[28] Etxegarai A., Eguia P., Torres E., Fernandez E. Impact of wind power in isolated power systems.

[29] Elsaharty M. A., Ashour H. A. Passive L and LCL filter design method for grid-connected inverters.

[30] Algaddafi A. (2016). *Advanced Modelling and Designing of an Optimal Controller Converter*.

